# Exclusive Breastfeeding Practice and Its Association among Mothers of under 5 Children in Kwango District, DR Congo

**DOI:** 10.3390/ijerph14050455

**Published:** 2017-04-25

**Authors:** Sarita Dhakal, Tae Ho Lee, Eun Woo Nam

**Affiliations:** 1 Yonsei Global Health Center, Yonsei University, Wonju, Gangwon-do 26493, Korea; saritadhakal1234@gmail.com (S.D.); leetaeh5@naver.com (T.H.L.); 2 Department of Health Administration, Graduate School, Yonsei University, Wonju, Gangwon-do 26493, Korea

**Keywords:** exclusive breastfeeding, breast milk, knowledge, DR Congo

## Abstract

The benefit of the breastfeeding has been well-established. In comparison to partial breast feeding, exclusive breastfeeding has even more benefits. The aim of this study was to identify the factors associated with breastfeeding exclusivity during the first 6 months of life in order to better target public health interventions in this community towards healthier infant nutrition and address child mortality in this population. A cross-sectional survey among 1145 random households was conducted in the Kwango district of the Democratic Republic of the Congo (DRC) during 2 November 2015 to 13 November 2015. Women of reproductive age from 15–49 years and having less than 5 years old child were selected for the study. Chi-squared test and bivariate and multivariate analyses were performed using SPSS. A major finding of this study is 49.2% of the mothers are exclusively breastfeeding their children, and marital status, literacy, place of delivery, knowledge of exclusive breastfeeding and access to radio are the key indicators for exclusive breastfeeding. Exclusive breastfeeding rate is almost equivalent to the national prevalence rate for the DRC. Providing adequate knowledge to raise awareness of exclusive breast feeding and increase involvement of health care providers in enhancing knowledge through antenatal care and during delivery and postnatal care will be the best approaches to increase exclusive breastfeeding practice.

## 1. Introduction

Breast milk is the ideal food for physical and mental growth and development of all infants. It contains all essential nutrients including carbohydrates, essential fats, proteins, minerals, and immunological factors [1]. Exclusive breastfeeding (EBF) means providing only breast milk to the infants; no other liquids or solids including water, except oral rehydration solution or drops/syrups of vitamins, minerals, or medicines [2]. Breast feeding is very important for public health and epidemiological studies because it has an important role in many different countries [3]. 

Breast milk is an ideal food which contains all the nutrients an infant needs for the first six months. Breastfeeding protects against diarrhea and common childhood illnesses such as pneumonia. It also has long term health benefits for the mother and child, such as reducing the risk of obesity in childhood [4] and adolescence. Breastfeeding has relationship with a higher intelligence quotient (IQ) in children [5,6]. There are significant benefits of EBF not only for infants but also for the mother and society [7]. Various researches have shown that breast milk is important for physical, neurological, and cognitive development of child that can reduces risks of allergies, infection, and non-communicable diseases during later stages of their development [8,9]. Therefore, breast feeding is considered as a cost effective infant-feeding method for families and society can reduce the risk of communicable and non-communicable childhood diseases [7,10]. A previous study showed that breast feeding practices in mother can protect against breast and ovarian cancer in them [9]. Global exclusive breast feeding rate to infants, younger than 6 months age, is less than 40%. Thus, one of the strategies of the Sustainable Development Goals is to increase exclusive breast feeding rate in under-five aged child to 50% [11].

Globally child health data shows that the Democratic Republic of the Congo (DRC) has the third highest child death in the world [12]. Under age 5 mortality is 104 per 1000 live births and infant mortality is 56 per 1000 live births [13]. DRC is a developing country with large assets of mineral resources and has potential for hydropower and agriculture development [14]. The national per capita income is U.S. $483.4 [15]. The average age of the mother among 25–40 age groups at birth of first child is 19.9 years. The demographic health survey shows that 14% women are in chronic under nutrition [13]. There exists a relation between breast feeding and maternal nutrition in case of chronic malnutrition in lactating mothers [16]. A study in Congo shows that Congolese mother gives additional foods to their infants, such as sugar syrup, water, tea, formula and porridge etc. [17]. Findings from epidemiological and biological studies showed that children who did not receive breast milk experienced major long-term adverse effects on health, nutrition and development [18]. A Lancet series on maternal and child undernutrition and obesity in low and middle income countries in 2013 showed that globally, an estimated 45% of all child deaths in 2011 were caused by malnutrition [19]. A study on breastfeeding behavior in Kinshasa, DRC showed that cultural belief is a factor that influences parents to give additional food other than human milk [18]. A study conducted in the DRC have found that breastfeeding practice is near-universal, but is not used exclusively [16]. A systematic review of the Nordic countries showed that EBF has several benefit thus breastfeeding should be enhancing to obtain long-term health benefit [20]. This study explored exclusive breastfeeding and its associated factors among mothers having under five children in Kwango District, DR Congo.

## 2. Methods

### 2.1. Study Design, Sampling and Statistical Analysis

The present study is a part of study conducted by Yonsei Global Health Centre, Republic of Korea in collaboration with a team from the School of Public Health, Kinshasa University, DRC for Korea International Cooperation Agency (KOICA) Maternal and Child Health (MCH) project in Kwango province. The population of the study consists of inhabitants of two Zone de Sante (Health Zones), Kenge and Boko, which are targeted by the MCH project in the province of Kwango. Target households included in the study are those with mother having at least one under 5 years old children and who agreed to participate in the household survey. At each Health Zone (Kenge and Boko), 30 health areas were selected which are supported by KOICA for the MCH project. This study used multi-stage cluster sampling method. In DRC, the numbers of households are divided into Health Areas relative to the population size. At each health area, we grouped villages into three strata: within 5 km far from the health center, located between 5 and 10 km far from the health center, located more than 10 km far from the health center. Then, one village was randomly selected from each of the above stratum. The number of households per village was selected proportional to the population size of each stratum. Households were selected by a random walk from a single entry point of the village. The entry points were selected by a simple random sampling and other samples were selected from the nearby houses until the total sample size complete in one stratum; and same process was repeated for the next stratum.

Sample size is calculated using “Raosoft” online software developed by Raosoft Inc., Seattle, WA, USA (Avaliable online: http://www.raosoft.com/samplesize.html) considering total population (290,000), margin of error of 3%, confidence interval of 95%, and the response distribution of 50%. The calculated sample size was 1064; and 8% of calculated sample (85) was added and it became 1149. Thus, a total of 1145 samples were collected for the study. The data were collected during 2 November 2015 to 13 November 2015. The collected information included household information with socio-demographics, water and sanitation, hand washing, access to mass media and use of information/communication technology, recognition of MCH service, fertility and birth history, maternal and newborn health, human immunodeficiency virus/acquired immunodeficiency syndrome HIV/AIDS, subjective health, post-natal health examination, and contraception. These factors were included to identify the factors associated with breastfeeding exclusivity during the first 6 months of life. In every respondent’s residence, a face-to-face interview was conducted to complete a questionnaire by Yonsei Global Health Center in collaboration with a team from the School of Public Health, Kinshasa University. The interview lasted for approximately one hour. A total of 19 enumerators were selected and adequately trained for survey. The questionnaire of this survey was obtained from Multiple Indicator Cluster Survey (MICS). Statistical analyses were performed using Statistical Package for Social Science (SPSS) version 21.0 (IBM Corp., Armonk, NY, USA). Proportion of EBF was computed. Bivariate and multivariate logistic regression analyses were used to examine the factors associated with the outcome variables of EBF. Infant until the age of 6 month is the period for EBF according to World Health Organization WHO [21]. EBF was measured using the following single question “Did you feed to your child only breast milk up to 6 months (180 days)?”.

### 2.2. Ethical Approval

Ethical approval for this study was obtained from the Institutional Review Board of Wonju Campus, Yonsei University (1041849-201406-BM-027-01) and the Université de Kinshasa, Ecole de Santé Publique (ESP/CE/021/2015) of the DRC. Informed consent was obtained from individual participants. 

## 3. Results

Among the 1145 women aged 15–49 years who had given birth in the past 5 years prior to this survey, 563 (49.2%) had practiced EFB. Out of all the women in the study, 39.3% were in the 26–35 years age group and among them 48.9% had practiced EBF. The 15–25 years age group consisted of 27% and 36 years and over was 15% of the total women in the study and among these groups, 45.2% and 49.4%, respectively, were providing EBF. Considering the education level, 42.1% of all mothers were literate and 55.1% of illiterate were providing EBF. The characteristics of the respondents and their association with EBF practice are shown in Table 1.

### Factors Related to Exclusive Breastfeeding

#### Bivariate Analyses 

Although cross-tabulation analyses showed significant relationships between selected variables (Table 1), we further analyzed all variables to test whether they were significant in univariate chi-squire test/correlation analysis by crude odds ratio (unadjusted) using logistic regression. Crude regression analysis showed that eight factors were significantly associated with EBF practice including marital status (odds ratio (OR) 2.21, OR at 95% confidence interval CI 1.54–3.15), literacy (OR 1.693 95% CI 1.33–2.14), antenatal care received during pregnancy (OR 5.426 95% CI 2.729–10.787), mothers with institutional delivery (OR 4.084 95% CI 2.73–6.09), delivery conducted by skilled health care personnel (OR 3.413 95% CI 2.262–5.150), postnatal care received after delivery (OR 2.656 95% CI 1.917–3.680), knowledge of EBF (OR 1.918 95% CI 1.513–2.431), and access to radio (OR 2.08 95% CI 1.58–2.74) (Table 2).

#### Multivariate Logistic Regression Analysis

We performed multivariate logistic Regression analysis, and presented adjusted odds ratio (AOR) and corresponding confidence intervals (CI). In this analysis, only five factors were significantly associated with EBF including marital status (AOR, 2.27; CI, 1.48 to 3.49), literacy (AOR, 1.40; CI, 1.04 to 1.88), mothers with institutional delivery (AOR, 8.42; CI, 1.86 to 38.02), knowledge of EBF (AOR, 1.78; CI, 1.33 to 2.38), and access to radio (AOR, 1.6; CI, 1.16 to 2.23).

## 4. Discussion

This cross-sectional study aimed to identify exclusive breastfeeding status and its associated factors among mothers having children younger than 5 years. The most important factors associated with EBF were found to be marital status, literacy, place of delivery, knowledge of EBF, and access to radio.

The prevalence of EBF in the study area is 49.2%, which is slightly higher than the global average rate of 40% [11] and national average rate of 48%.

The pattern of EBF in the current study showed that married mothers are more likely to practice EBF compared to unmarried or widowed mothers. This finding could be due to the support from family members to perform other household activities. A study in Myanmar revealed that family member support contributed to adherence to EBF by mothers [22]. Consistent with many previous studies [23,24,25], the logistic regression of this study indicated that literate mothers are more likely to practice EBF. Literate mothers can obtain knowledge from reading materials such as promotional pamphlets, broadcasting media and billboards. Utilization of health facility services also increases knowledge in the mother. The results of this study show that mothers who delivered their child at a health care facility are 8 times more likely to practice EBF. These results are similar to previous findings in Ethiopia, Tanzania and India. Studies in these countries revealed that literate women are three times more likely to practice EBF [25,26,27]. Knowledge is a basic factor in every field which is also supported by this study. Respondents with correct knowledge and awareness of EBF are more likely to practice EBF [28]. Radio is an easy and effective means of mass media. In many developing countries, there is evidence that health education and information via radio broadcasting can improve public health [29,30,31]. Thus, it is possible that information from radio broadcasting will have influence to mothers to practice EBF. In this study, respondent who had access to land was not statistically significant, but a study in People’s Republic of China suggested that household in high wealth quintile were less likely to provide exclusive breastfeeding within 6 months in comparison to those who were belong to low quantile groups [32].

Although antenatal care (ANC) and postnatal care (PNC) utilization is high in the study area, these two factors were not significantly associated with EBF. This lack of association could be due to insufficient knowledge and poor counseling techniques from the health personnel. Previous studies in the DRC indicated that adequate training for health care providers on breastfeeding support skills can aid in increasing the practice of EBF [14,19]. Studies have also shown that prenatal EBF intention was a strong predictor of EBF practice [33]. Therefore, skills improvement training might be helpful for health personnel to enhance their knowledge for better counseling while proving ANC and PNC. 

At last we would like to acknowledge for the question on explanatory variables which may bias to answer the respondents.

## 5. Conclusions 

EBF were statically significant with marital status, literacy, mother with institutional delivery, knowledge of EBF, and access to radio. Encouraging mother and family for EBF, and utilization of the community health worker for education may be the best approach. In this sample of Congolese families, better access to medical staff at delivery, and increased maternal education has the potential to improve infant feeding practice toward compliance with the WHO guidelines, and to save the lives of children, leading to enhanced economic prosperity and quality of life. Health education on EBF including information on techniques and advantages of EBF should be provided to mothers and their family. The ideal period for counseling mothers about EBF is the period during ANC and PNC. Thus, they could give health education while counselling mother during ANC and PNC. Also, in order to promote knowledge about benefit of EBF, it would be effective to broadcast from the radio. Promotion of EBF would be a best approach to prevent from undernourishment or malnourishment in child population where people cannot afford formula and nutrition supplement. EBF is healthy and cost effective methods of feeding.

## Figures and Tables

**Table 1:** General characteristics of the study population (n = 1145).

Variables	Exclusive Breastfeeding [n (%)]		Total [in Figure, (in %)]	*p*-Value
	Yes	No		
	563 (49.2)	582 (50.8)		
Age of the women				
15–25 years	140 (45.2)	170 (54.8)	310 (27.0)	0.533
26–35 years	220 (48.9)	230 (51.1)	450 (39.3)	
36 years and above	85 (49.4)	87 (50.6)	172 (15.0)	
Marital status				
Married	343 (55.1)	279 (44.9)	622 (54.3)	0.000
Other	220 (42.1)	303 (57.9)	523 (45.6)	
Access to land				
Yes	512 (51.8)	477(48.2)	989 (86.3)	0.000
No	51 (32.7)	105 (67.3)	156 (13.6)	
Literacy (can read and write)				
Yes	220 (42.1)	303 (57.9)	523 (45.6)	0.709
No	343 (55.1)	279 (44.9)	622 (54.4)	
Antenatal care received during pregnancy				
Yes	553 (51.1)	530 (48.9)	1083 (94.5)	0.000
No	10 (16.1)	52 (83.9)	62 (5.4)	
Place of delivery				
Institutional delivery	529 (53.4)	461 (46.6)	990 (86.4)	0.000
Home	34 (21.9)	121 (78.1)	155 (13.5)	
Delivery conducted by skilled health care personnel				
Yes	530 (52.5)	480 (47.5)	1010 (88.2)	0.000
No	33 (24.4)	102 (75.6)	135 (11.7)	
Postnatal care				
Yes	502 (53.3)	440 (46.7)	942 (82.2)	0.000
No	61 (30.0)	141 (70.0)	203 (17.7)	
Knowledge of exclusive breastfeeding				
Correct knowledge	363 (56.2)	283 (43.8)	646 (56.4)	0.000
Incorrect knowledge	200 (40.1)	299 (59.9)	499 (43.5)	0.000
Access to radio				
Listen every day	203 (61.3)	128 (38.7)	331 (31.0)	0.000
At least once per week	67 (44.1)	85 (55.9)	152 (14.2)	
Not at all	252 (43.2)	331 (56.8)	538 (54.8)	

**Table 2:** Bivariate and multivariate logistic regression analysis of exclusive breastfeeding.

Variables	Crude Odds Ratios (95% CI)	Adjusted Odds Ratios (95% CI)
Age of the women		
15–25 years	0.84 (0.58–1.22)	0.75 (0.50–1.13)
26–35 years	0.97 (0.68–1.39)	0.93 (0.63–1.37)
36 years and over	1	1
Marital status		
Married	2.21 (1.54–3.15) ***	2.27(1.48–3.49) ***
Other	1	1
Access to land		
Yes	0.953 (0.741–1.226)	1.07 (0.78–1.46)
No	1	1
Literacy (can read and write)		
Yes	1.693 (1.33–2.14) ***	1.40 (1.04–1.88) *
No	1	1
Antenatal care received during pregnancy		
Yes	5.426 (2.72–10.78) ***	1.76 (0.74–4.16)
No	1	
Place of delivery		
Institutional delivery	4.084 (2.73–6.09) ***	8.42 (1.86–38.02) **
Home	1	1
Delivery conducted by skilled health care personnel		
Yes	3.413 (2.26–5.15) ***	4.11(0.88–19.09)
No	1	1
Postnatal care		
Yes	2.656 (1.91–3.68) ***	1.32 (0.78–2.23)
No	1	1
Knowledge of EBF		
Correct knowledge	1.918 (1.51–2.43) ***	1.78 (1.33-2.38) ***
Incorrect knowledge	1	1
Access to radio		
Listen every day	2.08 (1.58–2.74) ***	1.6 (1.16–2.23) **
At least once per week	1.03 (0.72–1.48)	0.879 (0.57–1.34)
Not at all	1	1

Note: CI = Confidence Interval. EBF = Exclusive breastfeeding. The variables entered were marital status, literacy level, place of delivery, delivery conducted by skilled person and knowledge towards the EBF. *p*-value = *** <0.001, ** <0.01, * <0.05. Hosmer and Lemeshow value: Chi-square = 10.98, 0.20).
